# The evaluation of zebrafish cardiovascular and behavioral functions through microfluidics

**DOI:** 10.1038/s41598-021-93078-y

**Published:** 2021-07-05

**Authors:** Satishkumar Subendran, Yi-Chieh Wang, Yueh-Hsun Lu, Chia-Yuan Chen

**Affiliations:** 1 Department of Mechanical Engineering, National Cheng Kung University, No. 1 University Road, Tainan, 701 Taiwan; 2 Department of Radiology, Shuang-Ho Hospital, Taipei Medical University, New Taipei City, 235 Taiwan; 3 Department of Radiology, School of Medicine, College of Medicine, Taipei Medical University, Taipei, 110 Taiwan; 4 Department of Radiology, National Yang-Ming University School of Medicine, Taipei, 112 Taiwan

**Keywords:** Biomedical engineering, Mechanical engineering

## Abstract

This study proposed a new experimental approach for the vascular and phenotype evaluation of the non-anesthetized zebrafish with representative imaging orientations for heart, pectoral fin beating, and vasculature views by means of the designed microfluidic device through inducing the optomotor response and hydrodynamic pressure control. In order to provide the visual cues for better positioning of zebrafish, computer-animated moving grids were generated by an in-house control interface which was powered by the larval optomotor response, in conjunction with the pressure suction control. The presented platform provided a comprehensive evaluation of internal circulation and the linked external behaviors of zebrafish in response to the cardiovascular parameter changes. The insights from these imaging sections was extended to identify the linkage between the cardiac parameters and behavioral endpoints. In addition, selected chemicals such as ethanol and caffeine were employed for the treatment of zebrafish. The obtained findings can be applicable for future investigation in behavioral drug screening serving as the forefront in psychopharmacological and cognition research.

## Introduction

The recent advances in manufacturing technologies such as microfabrication, laser micromachining, and three-dimensional (3D) printing facilitated the microfluidic devices for immobilization, orientation, microinjection, flow-perfusion culture, and drug administration for small vertebrate animal tests such as zebrafish in the pharmaceutical industry. The successful use of these micro-fabricated devices was contributed by their effectiveness and robust nature. In-vivo toxicity and physiology testing on Lab-on-a-chip (LOC) represented a new path that can miniaturize and revolutionize. Numerously experimental studies involving behavioral, developmental, toxicology, and drug screening assays through the tests of larval zebrafish, were carried out on microfluidic and microwell devices, which were time-consuming and unfavorable for long-term imaging^1–3^. Due to the versatility of microfluidic devices incorporated with variously functional components, microfluidics has now proved to be a highly successful way of linking life science with micro- and nano-technology^4–7^.

Zebrafish (*D. rerio*) is one of the most common and beneficial vertebrate model organisms on which numerous biological developmental and disease studies have been carried out^8,9^. In addition to their use in fundamental studies, they were extensively employed in the fields of drug discovery, molecular toxicity, and therapeutics^10–12^. Zebrafish is a model vertebrate with a transparent appearance that made it possible to directly image developmental processes in-vivo. Zebrafish has become a popular animal model for developmental biology, pharmacology, and genetics due to specific advantages such as its highly genetic similarity to the human genome, rapid in vitro development with well-characterized developmental stages, easily observable transparent embryos, high fecundity, low-cost husbandry, and housing^13,14^. A zebrafish disease model in combination with automated microscopy techniques has enabled large-scale phenotypic whole organism screening assays, which has been established as a prominent technique for studying biological developmental processes to a greater extent within the complexity of the vertebrate organism^15,16^. However, there are still key challenges to be addressed before one can fully realize the potential of zebrafish in high-throughput screening approaches for various studies.

As high-resolution imaging and quantitative biology became increasingly important for developmental biologists, the zebrafish was established as a popular model organism for investigating various organ developments as it overcame the deficiencies that occurred in experimenting with the mammalian models (e.g. Rodents and Mice)^17–19^. Independent to which imaging system that was utilized, the scoring of accurate morphological was often complicated by the irregular orientation. Despite all the advances, several limitations of zebrafish more likely to be overcome were the precise orientation while imaging. Most commonly anesthetics and agarose gel are used for zebrafish immobilization in desirable positions. The use of anesthetics and agarose may lead to morphological damage as well as adversely affects the cardiac responses which are not suitable to evaluate behavioral and motor responses in zebrafish^20,21^. In addition, past researchers have documented several techniques of zebrafish larva immobilization and orientation within microfluidic platforms. For instance, Wittbrodt et al. used a 3D desktop printer to create orientation tools that could be used as molds for generating cavities in multi-wells filled with agarose enabling larvae to be positioned and imaged in either dorsal–ventral or lateral view^22^. Lin et al. have proposed a microfluidic "Fish-Trap" array device that could simultaneously immobilize and orient several zebrafish larvae allowing easy access and imaging of various organs for high-throughput analysis^23^. Chen et al. introduced a new microfluidic concept enabling precise and axial orientation control of zebrafish larvae by artificial actuation of cilia within the microchannel for biological screening^24^. Another microfluidic device proposed by Asal Nady et al. was capable of partially and reversibly immobilizing a zebrafish larva for quantitative behavioral assessment^25^. In order to investigate the feasibility of using a light-driven approach to induce optomotor response (OMR) motion in zebrafish larvae, a microfluidic system consisting of a chamber, a microwell, and three observation sections for imaging was developed by Mani et al^26^.

The larval zebrafish's optical clarity, high fecundity, rapid development, and small size conferred strong advantages in the field of high-throughput chemical screening^27–29^. Still, there are still certain drawbacks that need to be taken care of such as the difficulty of high-throughput screening in adult fish as well as inconsistent larval orientation. As mentioned above significant efforts have been made by the previous researchers, to address the need for zebrafish positioning/orientation with precise control and less detrimental effects, the current investigation interpreted the design and validation of a pioneered microfluidic technology by means of the synergistic effects based on the light pattern and hydrodynamic driving methods for their transportation and immobilization to the desired location. Such an arrangement can further facilitate the imaging need for internal blood circulation and external hydrodynamic behaviors for a comprehensive review of zebrafish responses in drug administration. As zebrafish is recognized as a reliable research model organism, the proposed method can significantly advance the current technology for the pharmaceutical sector through the employment of the zebrafish as an important model for evaluating novel drug candidates.

## Methods

### Design and fabrication of the microfluidics

**Figure 1:**
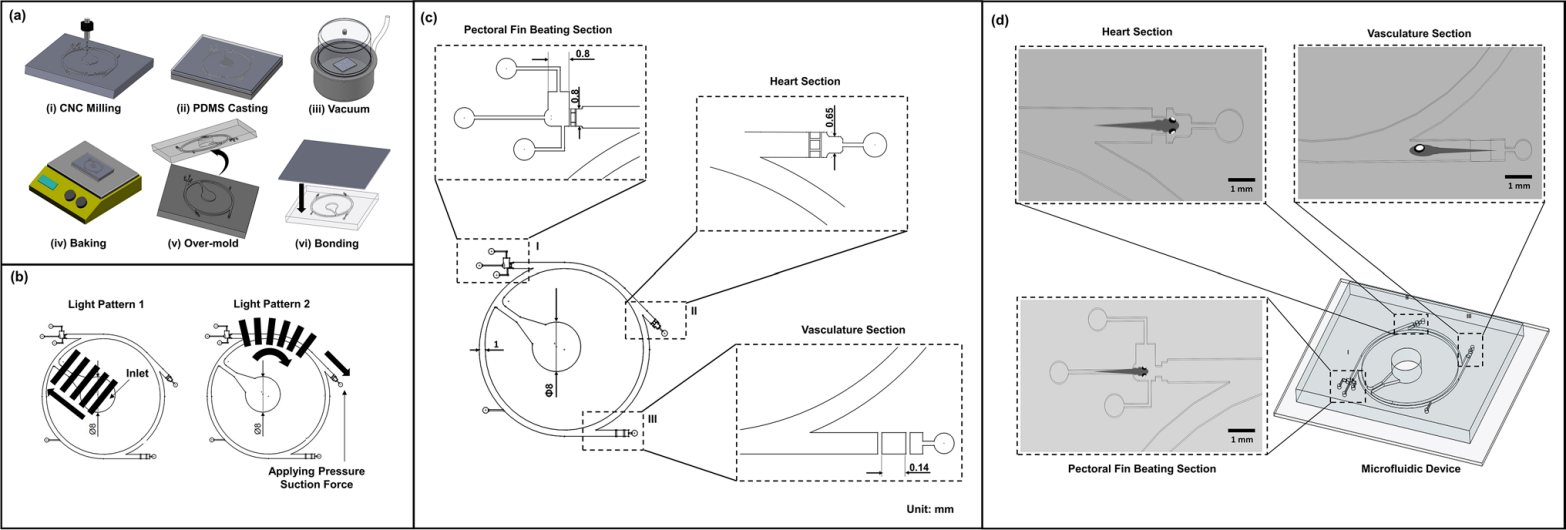
(**a**) Illustration of the series of processes (from I to vi) involved in the fabrication of the microfluidic device. (**b**) Illustration of moving directions of visual light patterns 1 and 2. (**c**) Illustration of geometric design details of various imaging sections in the microfluidic channel. (**d**) Illustration of various imaging sections—heart, Pectoral Fin Beating, and Vasculature.

To investigate various behavioral responses of the zebrafish within the microfluidic environment, a novel microfluidic device was designed based on hydrodynamic and optomotor response control for high throughput screening of the cardiovascular functional parameters in zebrafish. The microfluidic device was fabricated through a series of computerized numerical control micromachining techniques and polydimethylsiloxane (PDMS) casting processes. The microchannel design outline was drafted using commercial design software (Solid Works, Dassault Systems SolidWorks Corp., Waltham, MA, USA), and the design outline was imprinted on an acrylic substrate (5 mm thickness) using the CNC micromachining technique. In the fabrication process of the microfluidic device, a mixture of PDMS (Sylgard 184, Dow corning Corp., Midland, MI, USA) and curing agent in a ratio (10:1) was uniformly mixed, degassed in a vacuum chamber, and introduced into the acrylic mold. Further, the fabricated microfluidic structure was placed on a hot plate at a temperature of 90 °C for about 2 h for the curing process. As the final step, the PDMS channel was detached from the mold, and the inlet & outlet were punched through a biopsy puncher. The microchannel was subsequently bonded with another PDMS layer through an oxygen plasma process^30–32^. The principle was to make the microchannel and PDMS bond together to avoid fluid leakage^33^. The design details and the fabrication process are illustrated in Fig. 1a–c. The microfluidic chip was designed in such a manner to investigate various parameter modules such as heart rate, stroke volume, blood velocity, and behavioral activities in terms of pectoral fin beatings in transgenic zebrafish under three different Imaging Sections, namely, “Heart Section”, “Pectoral Fin Beating Section”, and “Vasculature Section”. Initial investigations were performed to assess the efficacy of the proposed system and to optimize the application parameters. To generate the OMR response in zebrafish larvae, moving gratings were generated by the LCD panel. An in-house-developed GUI was used to generate the moving gratings, and the moving gratings were provided along the direction of zebrafish larvae transportation to each of the imaging sections. The grating frequency and grating width ratio were set as 1.5 Hz and 1:1, respectively. To provide hydromechanical cues, an infuse/withdraw syringe pump was used, and the flow direction was determined according to the experimental purpose. Zebrafish larvae were positioned inside the inlet to test the behaviors of zebrafish larvae corresponding to the light and hydromechanical stimuli. The zebrafish larvae movements were recorded using a camera mounted on a tripod^34^. The design and dimensional details of both microfluidics are illustrated in Fig. 1.

### Zebrafish culture and chemical exposure

Transgenic zebrafish larvae Tg (gata1: DsRED) were used and their fertilized eggs were raised and cultured at the temperature ranging 28 °C ± 1 °C in a light: dark cycle of 14 h:10 h. This transgenic line expresses a red fluorescent protein (DsRED) signal from blood cells. A complete water change was carried out every 24 h ensuring water quality was maintained every day for each set of specimens for zebrafish larvae at 5, 7, and 9 days post fertilization (d.p.f.). The chemicals used in the study were ethanol and caffeine (CAS.39214 Alfa Aesar, UK) and their respective concentrations were chosen based on the existing literature^35–37^. The ethanol concentration considered (control and 3%) whereas two caffeine concentrations were chosen—25 mg/L (low), and 65 mg/L (high) along with the control group in order to examine the effects of concentration on cardiac functionalities and behavioral responses of zebrafish larvae (5, 7, and 9 d.p.f.). For the ethanol test, the exposure time was considered to be 10 min whereas for the caffeine test the exposure time considered was 15 min.

All experimental protocols were approved by the National Cheng Kung University Institutional Animal Care and Use Committee (IACUC) with the approval number: 109334 and all methods were carried out in accordance with relevant guidelines and regulations.

### Quantification of zebrafish heart beat

To obtain a clear image of the zebrafish heartbeat for quantification, the zebrafish body was immobilized through the geometry designs of the microchannel to reduce the movement of the zebrafish during the imaging. Under a stereomicroscope (Leica Z16 APO, Leica Camera AG, Wetzlar, Germany) equipped with a digital camera (Canon EOS 650D, Canon Inc., Tokyo, Japan), heartbeat images were recorded at 30 fps and then stored in a computer for further processing. The heartbeat images of the zebrafish were captured at 20 × magnification for 10 s and recorded under a light source to observe the blood flow changes in the heart (atrium or ventricle) for the estimation of the heartbeats. Since the zebrafish species used in this study was Tg (gata1: DsRED) which expressed red fluorescent protein (DsRED) in red blood cells. As a result, light and dark changes were observed in the zebrafish's heart during the contraction period. An open-source Tracker (Video Analysis) software (Open-Source Physics, OSP) was employed to analyze the areas with obvious light and dark changes and track the brightness value that changed over time. The obtained data were further processed using an inhouse MATLAB coding to perform Fast Fourier Transform (FFT) on the data to convert the time series signal into a Power Spectrum Density (PSD) where the peak value in the power spectrum corresponds to the heartbeat frequency of the zebrafish larvae. Among them, multiply this frequency by 60 to obtain the average heartbeats per minute (BPM). In short, the collected time-dependent data were processed in the frequency domain to identify the dominant frequency serving as the heartbeat information in a quantitative manner.

### High-speed imaging of zebrafish pectoral fins and the vein flow

In order to minimize the harmful effect on the larvae during the flow visualization measurement, the fluorescent particles were replaced by smashed egg yolk particles with a better biocompatible property^38^. The cooked egg yolk weighing 0.04 g was mashed thoroughly in 1.5 mL of DI water inside a conical tube. Further, a homogenous mixture of diluted egg yolk was prepared through the vortex mixer for minutes. The prepared egg yolk particle solution was infused into the designed microfluidic device for the quantitative observation of the induced fluid flow pattern disturbed by zebrafish pectoral fin beating. Images were obtained using an optical microscope (BX60, Olympus Corp., Japan) and a high-speed camera (NX4-S2, IDT, Tallahassee, FL, USA). The quantified flow fields were calculated by a particle image velocimetry (PIV) software package (Dynamic Studio, Dantec Dynamic A/S, Denmark), and the results are presented as the calculated velocity vectors overlapped with the vorticity contour map. An implemented adaptive PIV analysis method was employed with a grid step size of 16 × 16 (the vein flow) and 32 × 32 (induced flow by pectoral fins), the minimum Interrogation area (IA) size of 32 × 32, and the maximum IA size of 64 × 64. The first iteration used the largest IA size, while the subsequent iterations reduced the IA size. In order to justify the particle density and eliminated the background noise. Grid interpolation was used to increase the vector distribution and effectively reduce the bad vector and outliers. Required validation was also performed through the comparison between the experimental and analytical results.

### Statistical methods

To assess the impact of chemical exposure on zebrafish cardiovascular responses, a statistical algorithm was used to analyze the data. The factors affecting the final heartbeat results included the chemical exposure (ethanol and caffeine) and concentration together with the number of days after the fertilization of zebrafish eggs. The control group refers to zebrafish without chemical treatment. For data comparison, a two-tailed independent student’s t-test was selected to compare the significant difference between the control group and the chemical exposure group.

## Results

**Figure 2:**
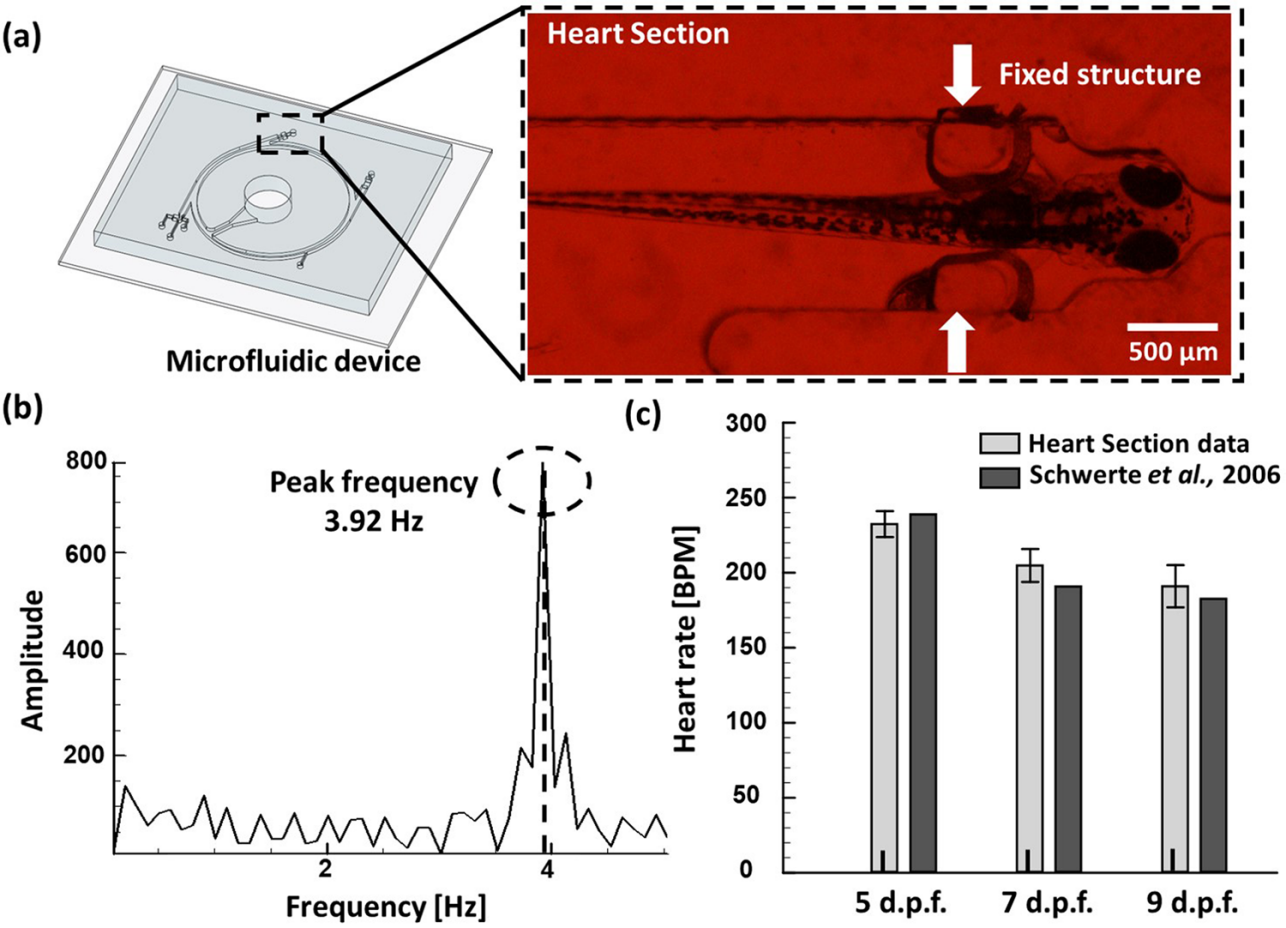
(**a**) Transportation of zebrafish larvae from the Inlet to the Heart Section through the proposed experimental platform. (**b**) Illustration of the dominant peak frequency spectrum. (**c**) Statistical data of the heartbeat rate of zebrafish (5, 7 and 9 d.p.f., N = 5 in each experimental group) obtained through the proposed method compared with the reference data of Schwerte et al^39^.

The fabricated microfluidic device was employed initially to investigate the representative parameters for cardiovascular and behavioral evaluation including the heart rate, blood velocity, and phenotype activities in terms of pectoral fin beatings and fin beats frequency of zebrafish (5, 7, and 9 d.p.f.) without exposure to any form of drugs. As depicted in Fig. 1d, all the three imaging sections were tested to verify that whether the transportation of zebrafish larvae was truly expressing a response to the generated gratings and was not due to the microfluidic design or natural innate behaviors of the zebrafish. From the experiment, it was observed that strong optomotor responses were evoked in all the zebrafish larvae corresponding to the movies with drifting grating patterns. In order to evaluate the cardiovascular functionality in the zebrafish within the proposed experimental platform, an initial test was conducted to perform heartbeat imaging by transporting the zebrafish to the respective imaging section (see Fig. 2a). An automated processing system (see “Quantification of zebrafish heartbeat” section) for the assessment of the zebrafish heartbeat rate was implemented after data acquisition. Figure 2b illustrates the dominant peak frequency which was further converted into the heartbeat rate. The statistical data of the heartbeat rate of zebrafish (5, 7, and 9 d.p.f.) are shown in Fig. 2c. The heartbeat rate of zebrafish (5, 7, and 9 d.p.f.) measured was 232.38 ± 8.65, 204.84 ± 10.96, and 190.98 ± 14.06 bpm, respectively. It was observed that the data obtained from the study were consistent with that of the control data from the reference. In general, the heartbeat rate increased with the developmental age until 5 d.p.f., and the heart rate decreased slowly as it developed into an adult zebrafish.

**Figure 3:**
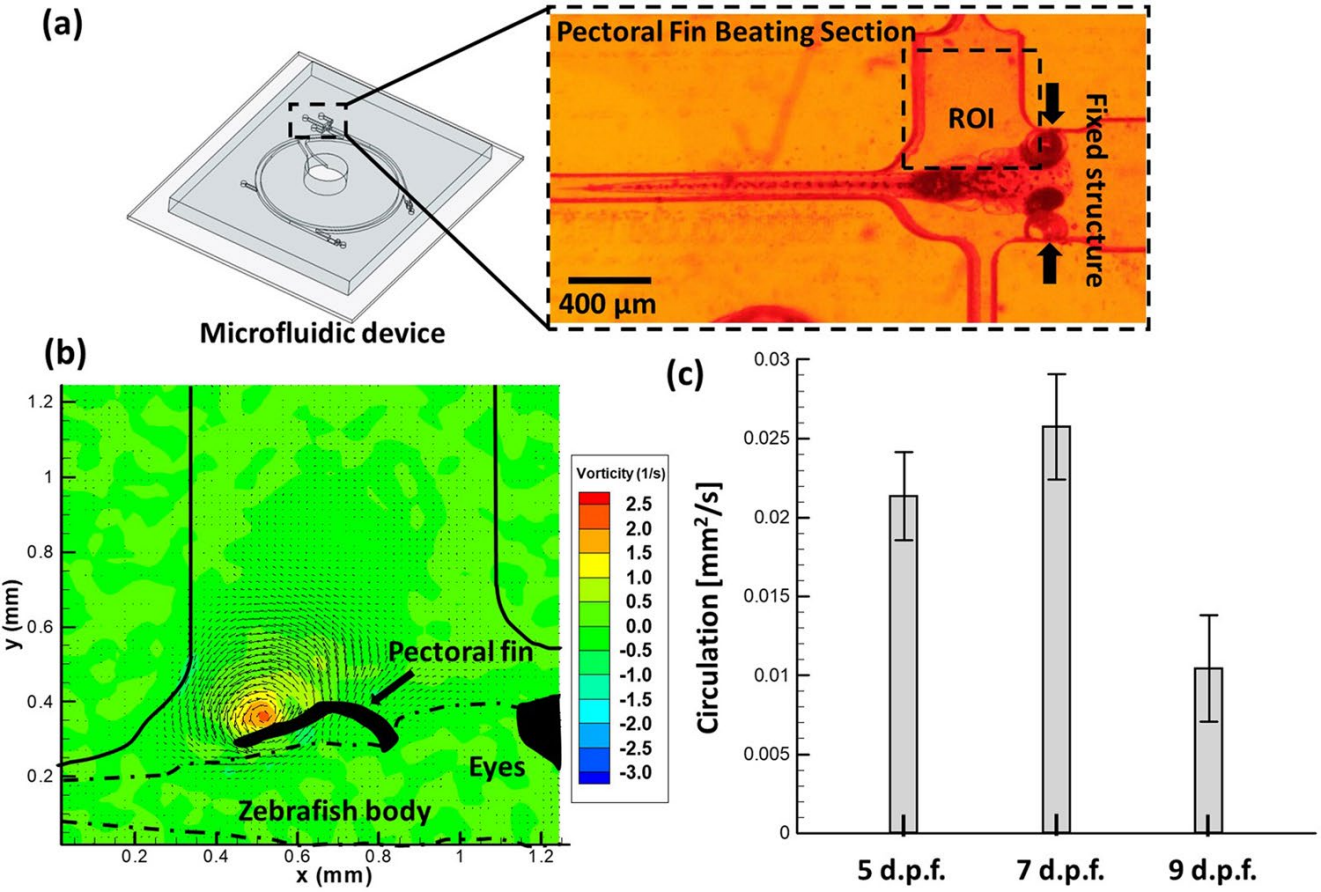
(**a**) Transportation of zebrafish larvae from the Inlet to the Pectoral Fin Beating Section through the proposed experimental platform. (**b**) Illustration of the generated vorticity during the pectoral fin beating in which the vorticity contour is super-imposed with the velocity vector field (black arrows) generated during pectoral fin beatings (**c**) Statistical data of the circulation formation induced by the beating of zebrafish (5, 7 and 9 d.p.f., N = 5 in each experimental group).

Consecutively, the behavioral changes in terms of the pectoral fin beating in normal condition without exposure to the drug was examined. Figure 3a illustrates the zebrafish within the pectoral fin beating section. Further, the hydrodynamic quantification was done to observe the generated vorticity during the pectoral fin beating (see Fig. 3b). The statistical data of the circulation generated during the pectoral fin beating of zebrafish (5, 7, and 9 d.p.f.) is shown in Fig. 3c. In addition, circulation (Γ) is defined as the line integral of the tangential component of velocity obtained around a closed curve in a flow field region^40^. The circulation generated due to the pectoral fin beating of zebrafish (5, 7, and 9 d.p.f.) measured as 0.0214 ± 0.0028, 0.0258 ± 0.0033, and 0.0104 ± 0.0034 mm^2^/s, respectively. The pectoral fin movements have been studied by previous researchers to examine their potential roles within the context of swimming performance and kinematics, and it is well known that exposure to drugs induced significant changes in heartbeat rate and swimming behaviors in zebrafish.

**Figure 4:**
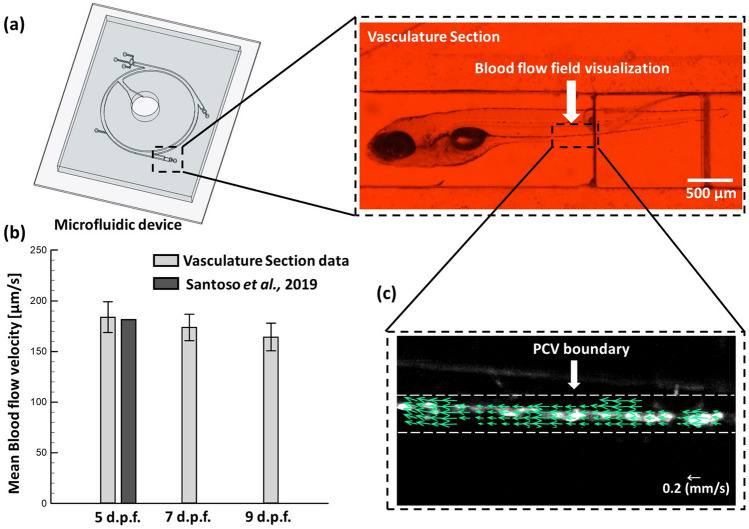
(**a**) Transportation of zebrafish larvae from the Inlet to the Vasculature Section through the proposed experimental platform. (**b**) Statistical data of the mean blood flow velocity of zebrafish (5, 7 and 9 d.p.f., N = 5 in each experimental group) compared with the reference data (5 d.p.f.) of Santoso et al^41^. (**c**) Illustration of the blood velocity vector map in posterior cardinal vein (PCV) region. Green arrows show the velocity of Red blood cells (RBCs).

As zebrafish is known to be an excellent model for studying the cardiotoxicity of a drug. To evaluate the blood flow velocity, a particular region of interest (ROI) was selected in the posterior cardinal vein (PCV) as it is one of the earliest developing blood vessels. Figure 4a illustrates the zebrafish within the vasculature section. Further, the velocity measurement was done (see High-speed Imaging of Zebrafish pectoral fins and the vein flow) to observe the blood flow within the PCV region. The mean blood flow velocity measured was 183.8 ± 15.1, 173.8 ± 13.1, and 164.2 ± 13.6 µm/s for zebrafish (5, 7, and 9 d.p.f.) respectively. As illustrated in Fig. 4b, a gradual decrease in the trend was observed in the mean blood flow velocity of zebrafish (5, 7, and 9 d.p.f.). Further, Fig. 4c. illustrates the Mean blood velocity vector map of the PCV region. Still, the knowledge linked to the cardiovascular functions and external swimming behaviors through the drug effects required more attention. The presented results provided the benchmark for further identifying the crosslinks among these intrinsic factors and served as a comprehensive example for animal test assessment for new drug development.

**Figure 5:**
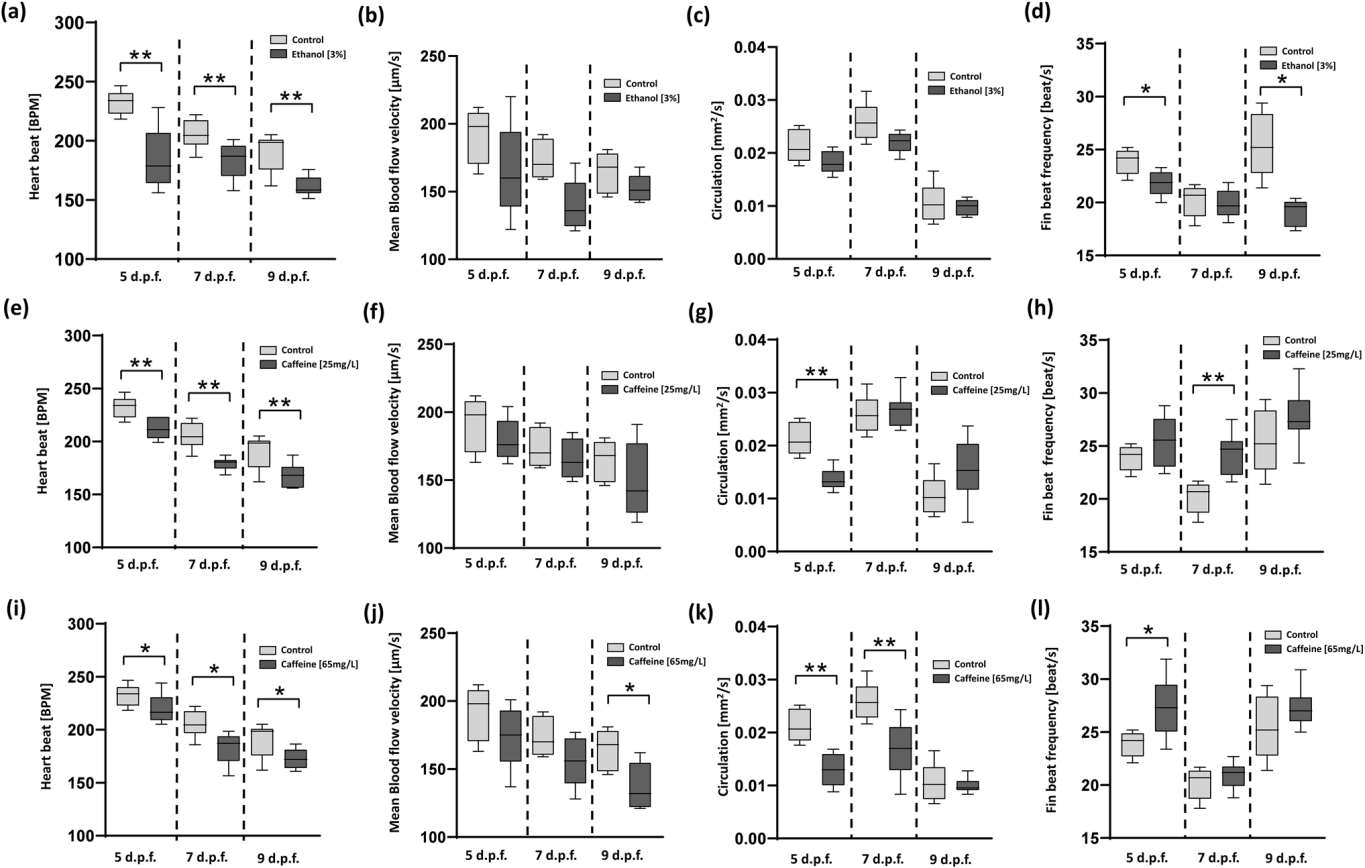
The High-throughput screening of parameters including heartbeat rate (N = 10 in each experimental group), mean blood flow velocity (N = 5 in each experimental group), circulation formation induced by the pectoral fin beating (N = 5 for control and ethanol, N = 10 for control and caffeine), and fin beat frequency (N = 5 for control and ethanol, N = 10 for control and caffeine) of zebrafish larvae (5, 7, and 9 d.p.f.) in response to the drug treatments (ethanol and caffeine) by transporting to the respective imaging sections. (**a**) Heartbeat rate, and (**b**) mean blood flow velocity (**c**) circulation and (**d**) fin beat frequency of zebrafish larvae in response to ethanol (3.00%). (**e**) Heartbeat rate, and (**f**) mean blood flow velocity (**g**) circulation and (**h**) fin beat frequency of zebrafish larvae in response to caffeine (25 mg/L), whereas (**i**) Heartbeat rate, and (**j**) mean blood flow velocity (**k**) circulation and (**l**) fin beat frequency of zebrafish larvae in response to caffeine (65 mg/L) respectively. Results that are significantly different from the control are indicated by asterisks (*p < 0.05, **p < 0.01).

Treatment of ethanol was employed in this study because of its significant effect on the cardiovascular physiology and developmental parameters in zebrafish larvae as well as in other model organisms that have been previously studied by past researchers^42–44^. It was observed that exposure to ethanol resulted in a significant drop (from 232.28 ± 8.65 to 185.88 ± 23.94 bpm, 204.84 ± 10.96 to 183.18 ± 11.11 bpm, and 190.98 ± 14.06 to 161.28 ± 7.50 bpm) (**p < 0.01) in zebrafish larvae’s heartbeat (5, 7, and 9 d.p.f.) as statistically illustrated in the Fig. 5a. This significant drop in the heart rate was obvious as per previous studies. Further, the study was extended to investigate the corresponding mean blood flow velocity in response to the ethanol treatment. The measured values are 165.2 ± 31.6, 139.6 ± 17.2, and 152.2 ± 9.1 µm/s for zebrafish (5, 7, and 9 d.p.f.) respectively (see Fig. 5b). The results illustrated no significant changes with a downward trend as compared to the control group. These cardiovascular changes induce significant behavioral changes in terms of the pectoral fin beating. In the absence of treatment, a forward vortex flow was generated around the pectoral fin region in the power stroke period. This fin beating cycle generates vorticity around the surrounding region which was quantified hydrodynamically using a micro-PIV tool. The quantified data was further used to evaluate the circulation generated due to the pectoral fin beating.

Figure 5c illustrates the data representing the circulation generated by zebrafish larvae (5, 7, and 9 d.p.f.) exposed to the ethanol treatment. The circulation generated due to the pectoral fin beating of zebrafish (5, 7, and 9 d.p.f.) control group compared to the ethanol treatment is measured as 0.0214 ± 0.0028 to 0.0183 ± 0.0019 mm^2^/s, 0.0258 ± 0.0033 to 0.0221 ± 0.0018 mm^2^/s, and 0.0104 ± 0.0034 to 0.0097 ± 0.0014 mm^2^/s respectively. Further, fin beat frequency of zebrafish (5, 7, and 9 d.p.f.) in response to the ethanol treatment was measured. The measured findings are 21.8 ± 1.1, 19.9 ± 1.2, and 19.0 ± 1.1 (see Fig. 5d). Thus, from the fin beat frequency data it can be observed that the ethanol treatment significantly hindered those in zebrafish 5 and 9 d.p.f. (*p < 0.05). These complex changes in the pectoral fin beats indicated that by impairing motor coordination, ethanol could alter even the swimming characteristics of ethanol-treated zebrafish larvae.

Caffeine was accepted to act as a stimulant on the central nervous system, it is worth noting that in fact, this drug exerts distinct responses depending on the amount used. Caffeine has a biphasic effect to stimulate locomotion and decrease anxiety at low levels, and diminish activity and increase anxiety-like behavior at doses above 50 mg/L. According to the literature, different doses of caffeine will have different effects^36,45^. Thus, to investigate the insights of the cardiac functional responses of zebrafish larvae towards exposure to higher (65 mg/L) and lower (25 mg/L) concentrations of caffeine was employed in the present study. Firstly, in the case of a lower concentration of caffeine, it was observed that there was an obvious decrease in the heartbeat rate of zebrafish larvae. The measured heartbeat rate of zebrafish (5, 7, and 9 d.p.f.) are 212.34 ± 8.86, 179.04 ± 5.48, and 167.94 ± 10.09 bpm as illustrated in Fig. 5e. As illustrated in Fig. 5f, there was no major changes observed in the mean blood velocity data. Although the result showed a downward trend (for both heartbeat and mean blood velocity), the concentrations used in this study did not cause a major impact.

Further, the observed corresponding circulation generated due to the pectoral fin beating of zebrafish (5, 7, and 9 d.p.f.) are 0.0139 ± 0.0019, 0.0267 ± 0.0029, and 0.0154 ± 0.0053 mm^2^/s, respectively as illustrated in Fig. 5g. It was observed that zebrafish (5 d.p.f) had a significant decrement (**p < 0.01) in the circulation. In addition, that although there was no significant difference in zebrafish (7 and 9 d.p.f), but there was an upward trend. This phenomenon may be due to a fact that at 9 d.p.f., the growth of larvae fish tended to be mature, and the cardiovascular development were relatively completed resulting that caffeine (25 mg/L) provided a stimulating effect on zebrafish, due to which circulation was enhanced^45^. Further, the fin beat frequency of zebrafish (5, 7, and 9 d.p.f.) in response to the caffeine treatment (25 mg/L) was measured. The measured findings are 25.4 ± 2.2, 24.4 ± 1.8, and 27.8 ± 2.3 (see Fig. 5h). There was significant increment (**p < 0.01) observed in the zebrafish (7 d.p.f.). Although there was no significance in zebrafish (5 and 9 d.p.f), but there was a gradual increase due to the stimulating effect of caffeine.

Secondly, in the case of a higher concentration of caffeine, the measured heartbeat rate of zebrafish (5, 7, and 9 d.p.f.) are 219.66 ± 12.67, 182.71 ± 14.33, and 172.26 ± 8.68 bpm as illustrated in Fig. 5i. The trend of decline in heartbeat rate for higher concentration was the same as that of the low concentration of caffeine. Compared to the lower concentration, the drop was lower in the higher concentration of caffeine. This may be because the difference in concentration is not too significant. As illustrated in Fig. 5j, there was a significant drop in in the mean blood velocity in zebrafish (9 d.p.f.). The corresponding circulation generated due to the pectoral fin beating of zebrafish (5, 7, and 9 d.p.f.) are 0.0130 ± 0.0028, 0.0169 ± 0.0049, and 0.0100 ± 0.0014 mm^2^/s, respectively as illustrated in Fig. 5k. The trend of the circulation observed in the case of higher concentration is different from the low concentration, indicating a significant downward trend in zebrafish (5 and 7 d.p.f.) as compared to the control group (*p < 0.01). The measured findings for fin beats frequency are 27.5 ± 2.6, 20.9 ± 1.2, and 27.3 ± 1.8 (see Fig. 5l). There was significant increment (*p < 0.05) observed in the zebrafish (5 d.p.f.).

## Discussion

The major objective of this work was to transport the zebrafish larvae efficiently within the microfluidic environment using the proposed experimental platform to all the three imaging sections for screening, and this was carried out by regulating the OMR behavioral response^46^ of the zebrafish using the moving grating of the LCD panel at a temporal frequency of 1.5 Hz in conjunction with the pressure suction control. OMR is one of the visuomotor responses seen in zebrafish wherein they follow the translational whole-field motion to stabilize their position in the flow field and swim along the direction of the motion^26^.Through the inlet, a single zebrafish was loaded into the microfluidic channel via a loading capillary. Further, the light pattern was turned ON to transport the zebrafish to the circular region. As soon as the zebrafish reached the circular region, the light pattern was changed by indicating a new path for the zebrafish to move forward. Once, the zebrafish reached the desired imaging section I, the pressure suction control was carried out through a syringe pump to transport the zebrafish to the respective imaging sections. The patterns of the moving gratings were designed in such a way that the zebrafish can be transported to all the imaging sections sequentially (see Supplementary Video S1) and can be integrated with the microscope stage. In addition, the visual and hydromechanical cues were regulated accordingly during the experimental process while transporting the zebrafish to each imaging sections in a well-organized manner. Thus, the investigation was performed using the zebrafish (control) to evaluate all the three parameters (heart beat rate, mean blood velocity, and circulation). With this design the platform can be further integrated into an existing microscope setup with user friendly interface to facilitate the biologists for performing the tasks.

Further, to extend the suitability of the fabricated device to investigate drug toxicity, high-throughput screening was performed to evaluate the cardiovascular functions and behavioral responses in zebrafish larvae (5, 7, and 9 d.p.f.) by drug treatments (ethanol and caffeine). The experiment was performed by successfully immobilizing the zebrafish in the respective sections (heart, pectoral fin beating, and vasculature sections) within the microfluidic chip. The focus was to investigate the linkage between significant heart beat changes and the predominant changes in behavioral responses (in terms of pectoral fin beating) of zebrafish larvae. The findings illustrated (see Fig. 5a–d) validated that ethanol-induced cardiovascular heart beat changes had a substantial impact on the behavioral responses in terms of the pectoral fin beating. Previous studies have been concluded that ethanol induces hyperactivity at concentrations below 2% and hypoactivity at concentrations above 4% respectively^47,48^. It is considered that exposure concentration is one of the most significant experimental parameters in toxicity studies.

In past studies, the concentrations of caffeine used were relatively higher which resulted in zebrafish’s heartbeat to completely stop. Caffeine is known to cause calcium release into the cytoplasm from intracellular stores by activating ryanodine sensitive receptors on the sarcoplasmic reticulum^49,50^. Additionally, calcium plays a key role in muscle contraction, synaptic transmission and cardiac neurotransmission^51,52^. It is hypothesized that the possible mechanism of caffeine’s effect on zebrafish’s heartbeat is that changing concentration of calcium indirectly can lead to the transmission of cardiac nerves and thus reduce the heartbeat. According to the literature, a higher concentration of caffeine can cause anxiety and stress^36^. This may be one of the reasons leading to a decline in the circulation generated by the pectoral fin beating of zebrafish and incline in the fin beats frequency (see Fig. 5k and 5l). Caffeine impacted the functioning of the central nervous system when it exceeded the therapeutic range. As such, when the dosage was too low it still caused an impact whereas for higher dosage it led to intoxication. In this context, past studies indicated conflicting behavioral effects to exposure of caffeine in zebrafish such as both increases in locomotor activity and decrease in motor response were observed.

The limitation of the presented devices/methods is delineated in the following. First, with the present design the screen can only be done by a single zebrafish larva in each imaging section but it can be resolved if the present platform can be connected in a sequential manner for the batch processing. In addition, Zebrafish larvae used in the proposed system required a well-developed OMR responsive specimen. Thus, only zebrafish with 4 d.p.f. or order can be used to perform the investigations. On top of it several manual steps are required in the currently setup including the loading of the zebrafish. Still such limits can be overcome for the inclusion of the autonomous arrangement.

## Conclusion

This study discussed comprehensively a new experimental approach for the vascular and phenotype evaluation of the non-anesthetized zebrafish (5, 7, and 9 d.p.f.) with representatively imaging orientations for heart, pectoral fin beating, and vasculature views through the designed microfluidic device through inducing the optomotor response and hydrodynamic pressure control. A detailed assessment of the internal circulation (i.e., cardiac changes) and associated external actions (i.e., behavioral responses) of zebrafish was investigated. As a proof-of-concept demonstration, a chemical exposure assay using ethanol (3%) and caffeine (25 and 65 mg/L) was employed to evaluate the heartbeat changes, mean blood velocity, time-averaged circulation generated due to pectoral fin beating and fin beats frequency of zebrafish (on-chip) respectively. Exposure to ethanol led to an obvious downward trend in the heartbeat rate and mean blood flow velocity. These cardiovascular changes in zebrafish induced behavioral changes such as circulation and fin beats frequency. It was observed that the cardiovascular changes significantly hindered the behavioral responses of zebrafish at various developmental stages (5, 7, and 9 d.p.f.). A similar trend was observed for heart beat in the case of caffeine (25 mg/L), but the drop was considerably higher than the exposure to ethanol. There were no major changes observed in mean blood flow velocity. But it was observed that at early developmental stage (5 d.p.f) had a significant drop in the circulation whereas at later stages (7 and 9 d.p.f), there was a rise in the circulation. On contrast to the circulation, fin beats frequency of zebrafish increased gradually due to the stimulating effect of caffeine. In the case of caffeine (65 mg/L) higher concentration exposure reduced the heart beat as well as hindered the pectoral fin movements significantly as higher caffeine doses induced anxiety-like behavior and reduced the locomotion in zebrafish. Additionally, the mean blood flow velocity was decreased. The resulted mean blood velocity was relatable due to the significant effects in the heartbeat rate of zebrafish. As caffeine exposure cause anxiety and stress in zebrafish, this led to decline in the circulation generated by the pectoral fin beating of zebrafish and incline in the fin beats frequency. The insights from these imaging parts have been expanded to identify the connection between cardiac parameters and behavioral endpoints. Thus, the proposed microfluidic system is capable of operating autonomously and multifunctionally for the evaluation of zebrafish in a fast and non-invasive manner. The relevance, feasibility, and suitability of the proposed experimental approach have been validated successfully. This paradigm is suitable for rapid screening and can provide considerably more information on the behavioral effects of toxicants and can be used for novel drug testing as well as for behavioral screening.

## Supplementary Information

Supplementary Video 1.
